# Supplementary material for: Modular metabolite assembly in Caenorhabditis elegans depends on carboxylesterases and formation of lysosome-related organelles
Source: eLife. 2020 Oct 16;9:e61886. doi: 10.7554/eLife.61886 (PMC7641594; doi:10.7554/eLife.61886)
Supplement: Supplementary file 1. — NMR spectra of synthetic intermediates and newly identified metabolites. [file elife-61886-supp1.pdf]

NMR Spectra Appendix

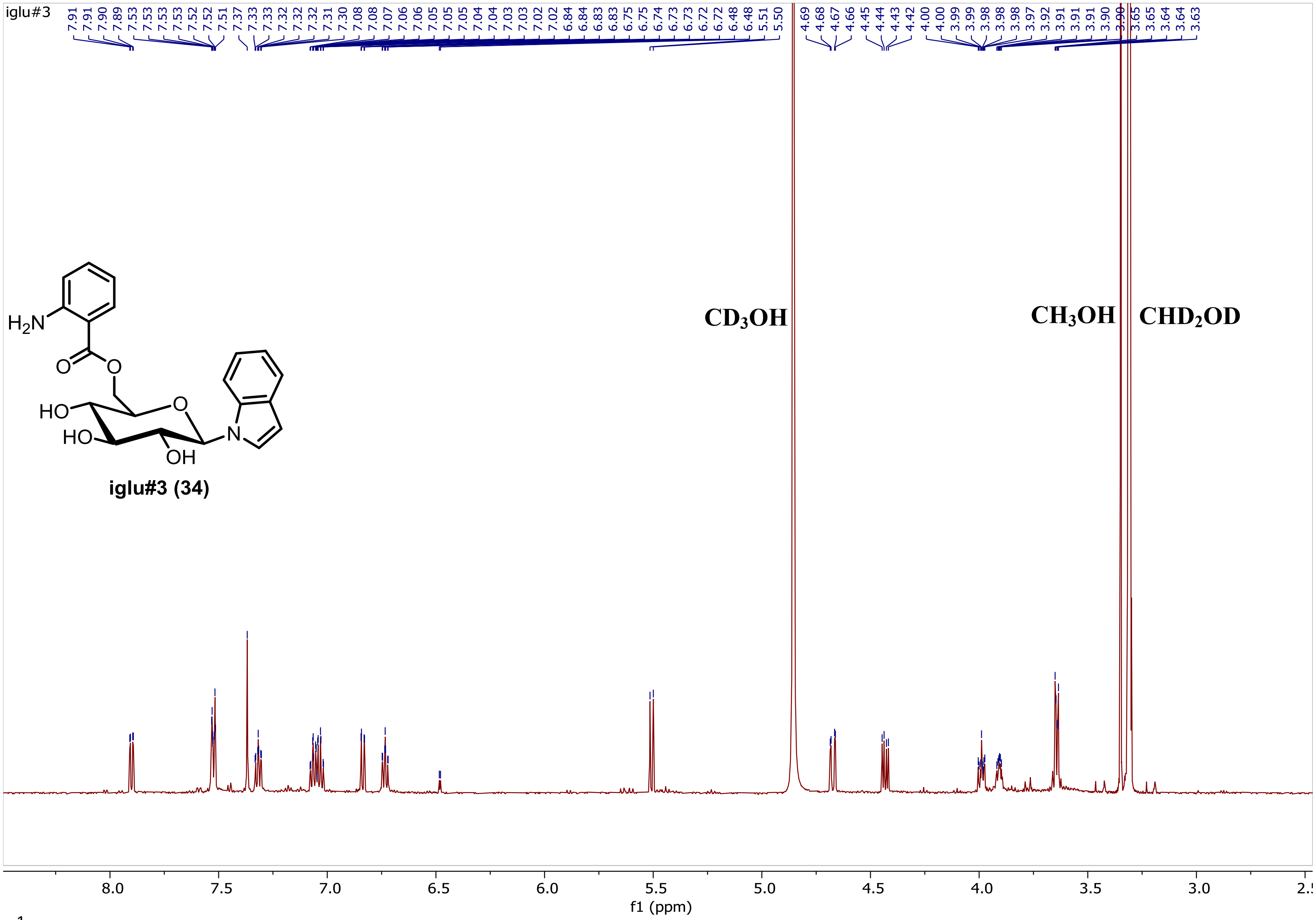

^{1}H NMR spectrum (600 MHz) of iglu#3 (**34**) in methanol-d_4.

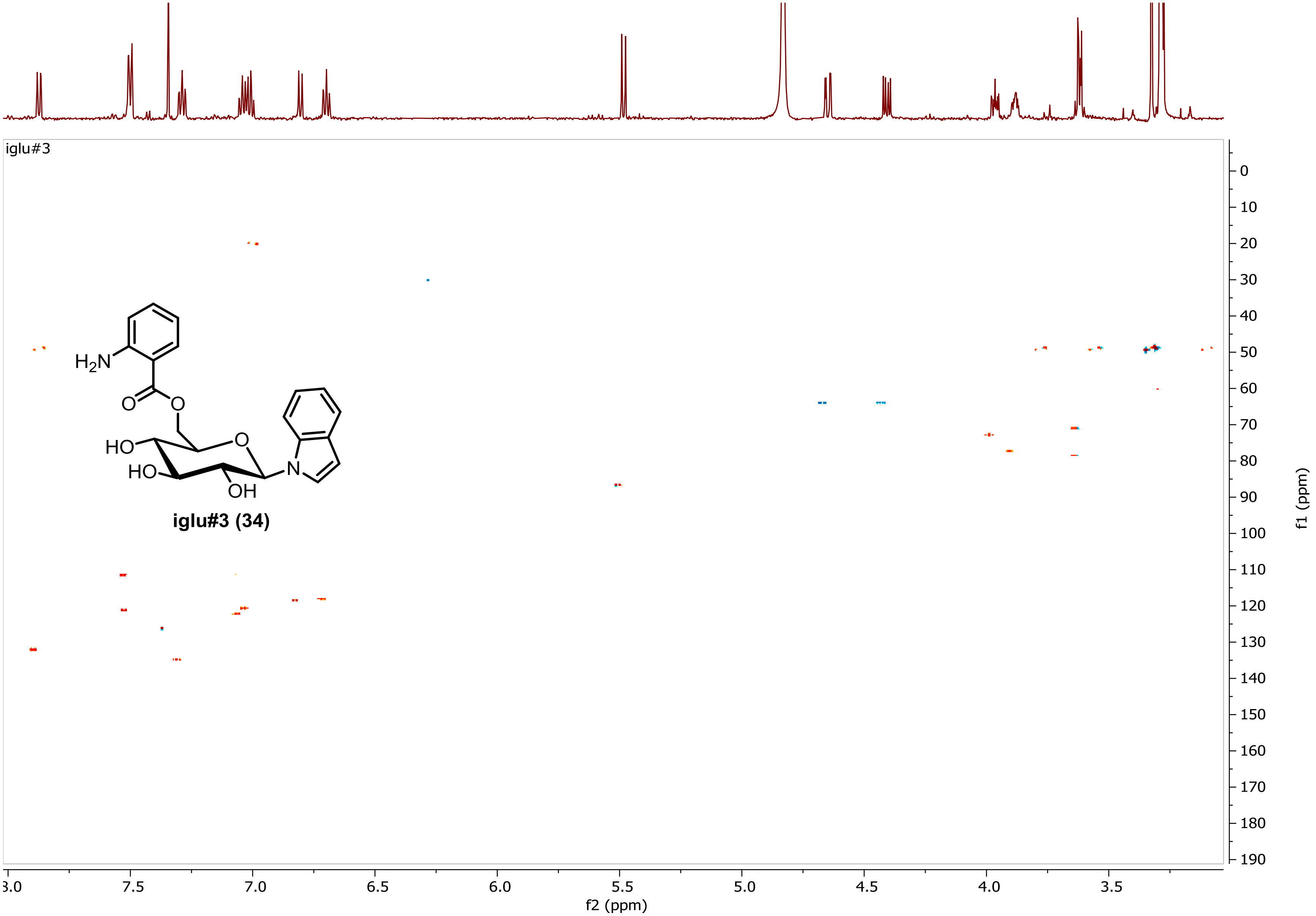

HSQC spectrum (600 MHz) of iglu#3 (**34**) in methanol-d_4.

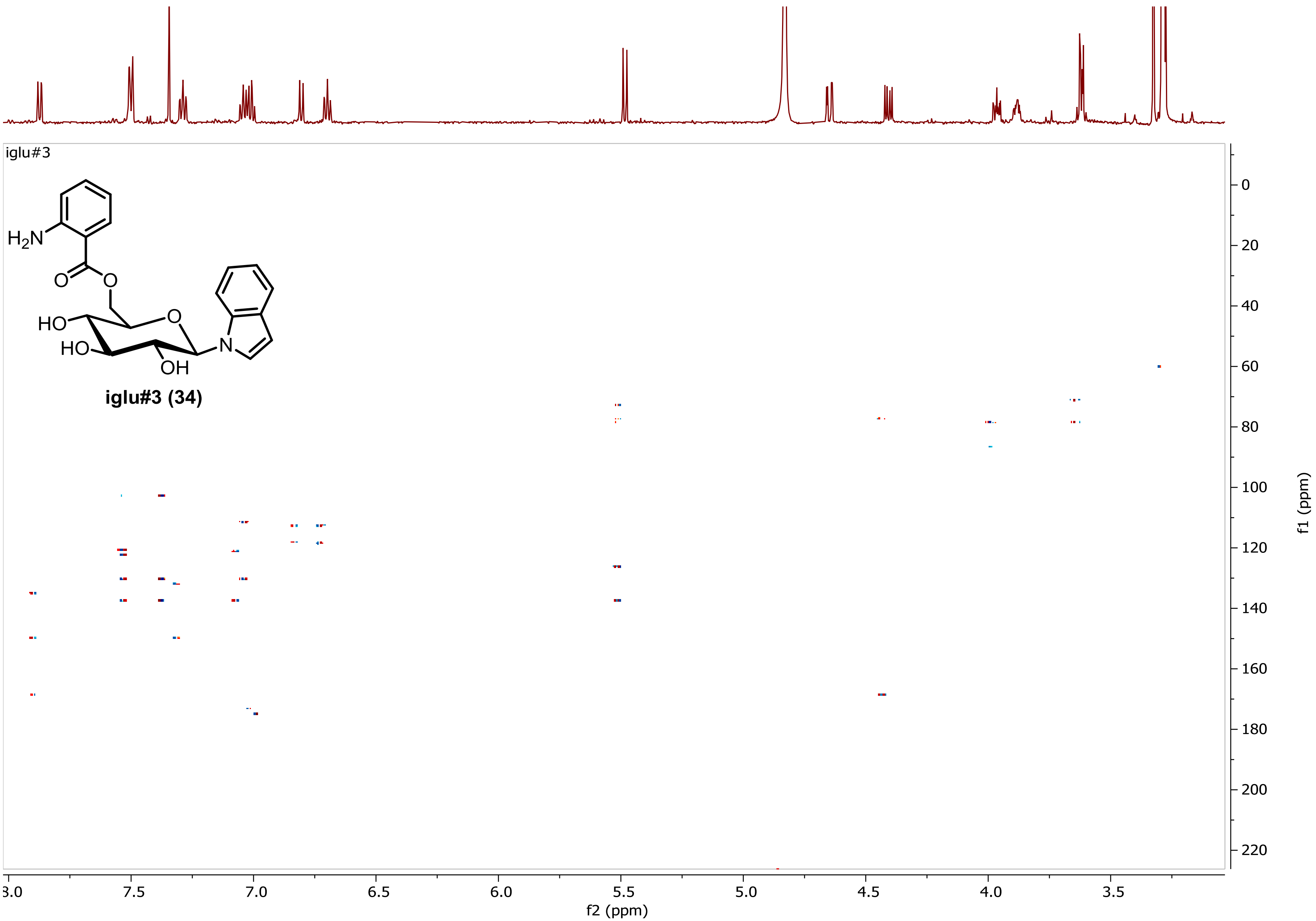

HMBC spectrum (600 MHz) of iglu#3 (**34**) in methanol-d_4.

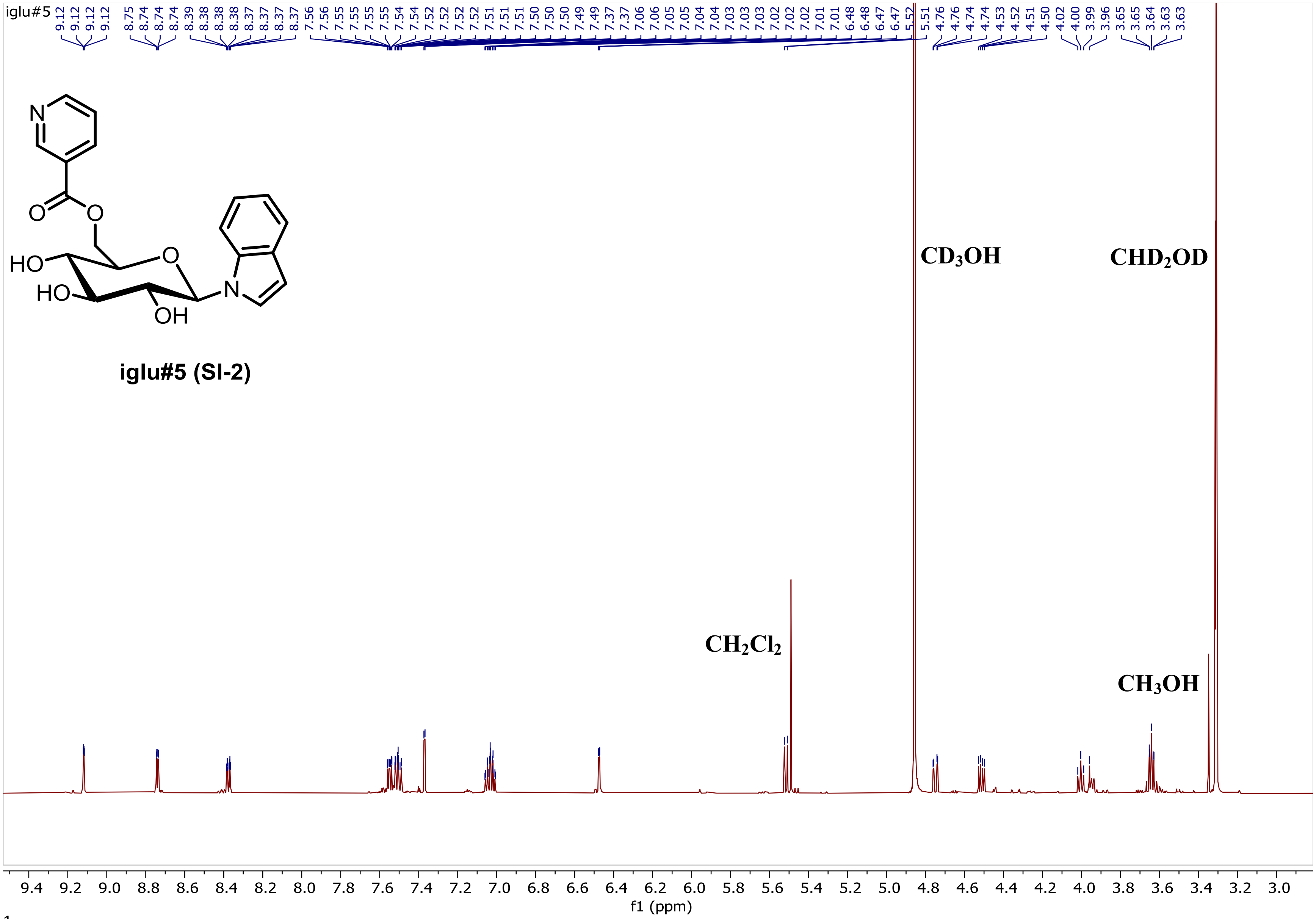

iglu#5 (SI-2)

^{1}H NMR spectrum (600 MHz) of iglu#5 (**SI-2**) in methanol-d_4.

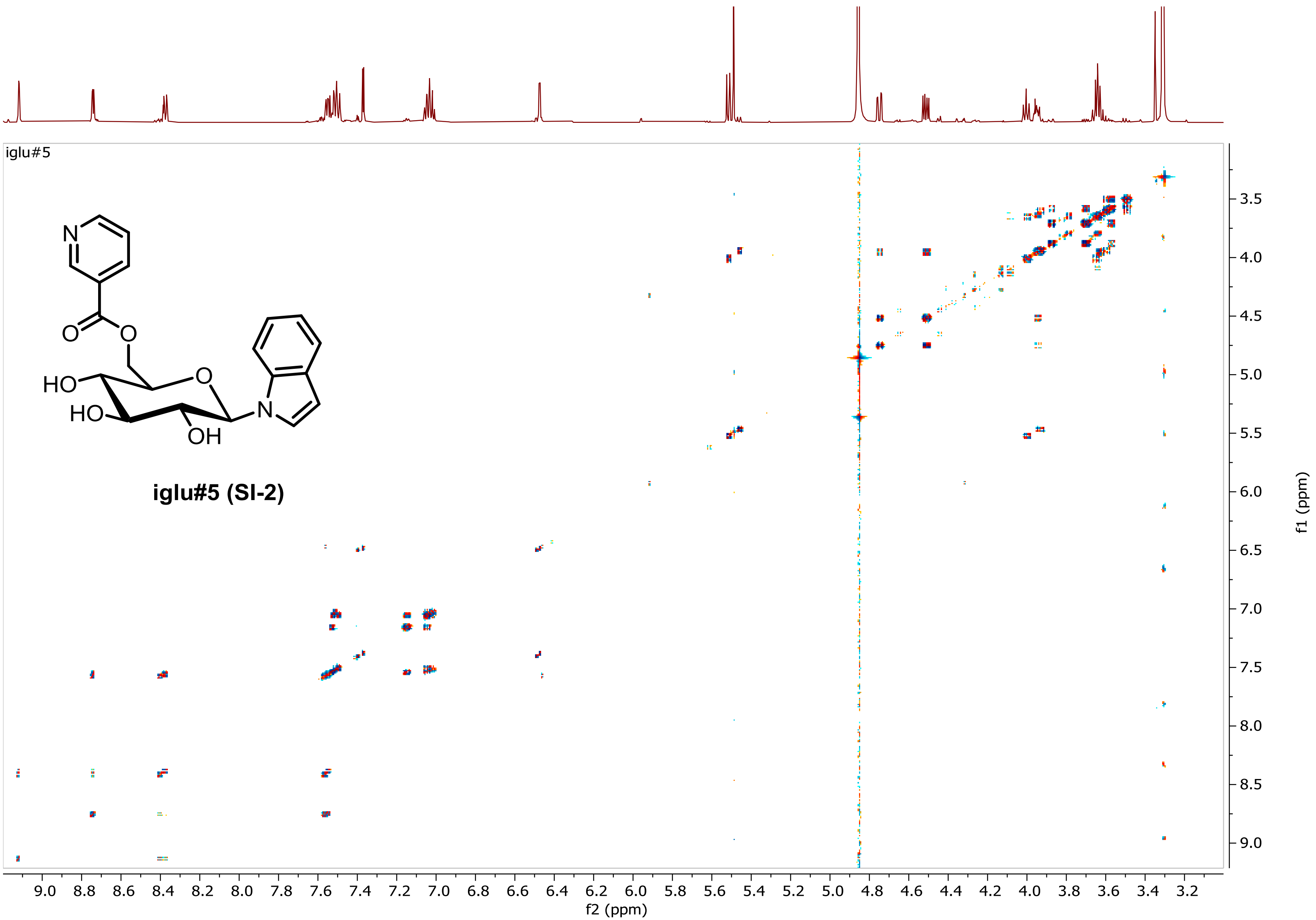

dqfCOSY spectrum (600 MHz) of iglu#5 (**SI-2**) in methanol-d_4.

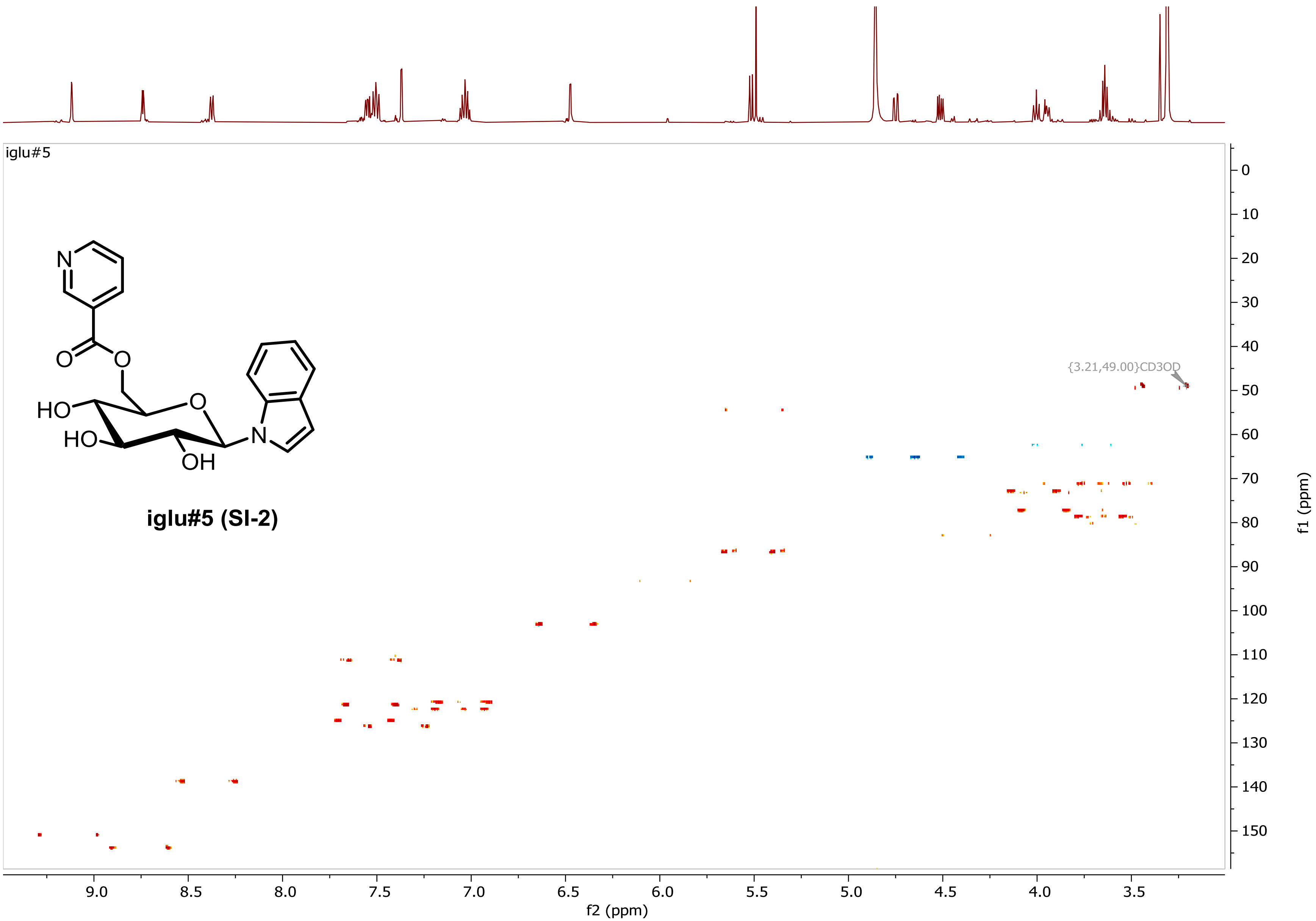

HSQC spectrum (600 MHz) of iglu#5 (**SI-2**) in methanol-d_4.

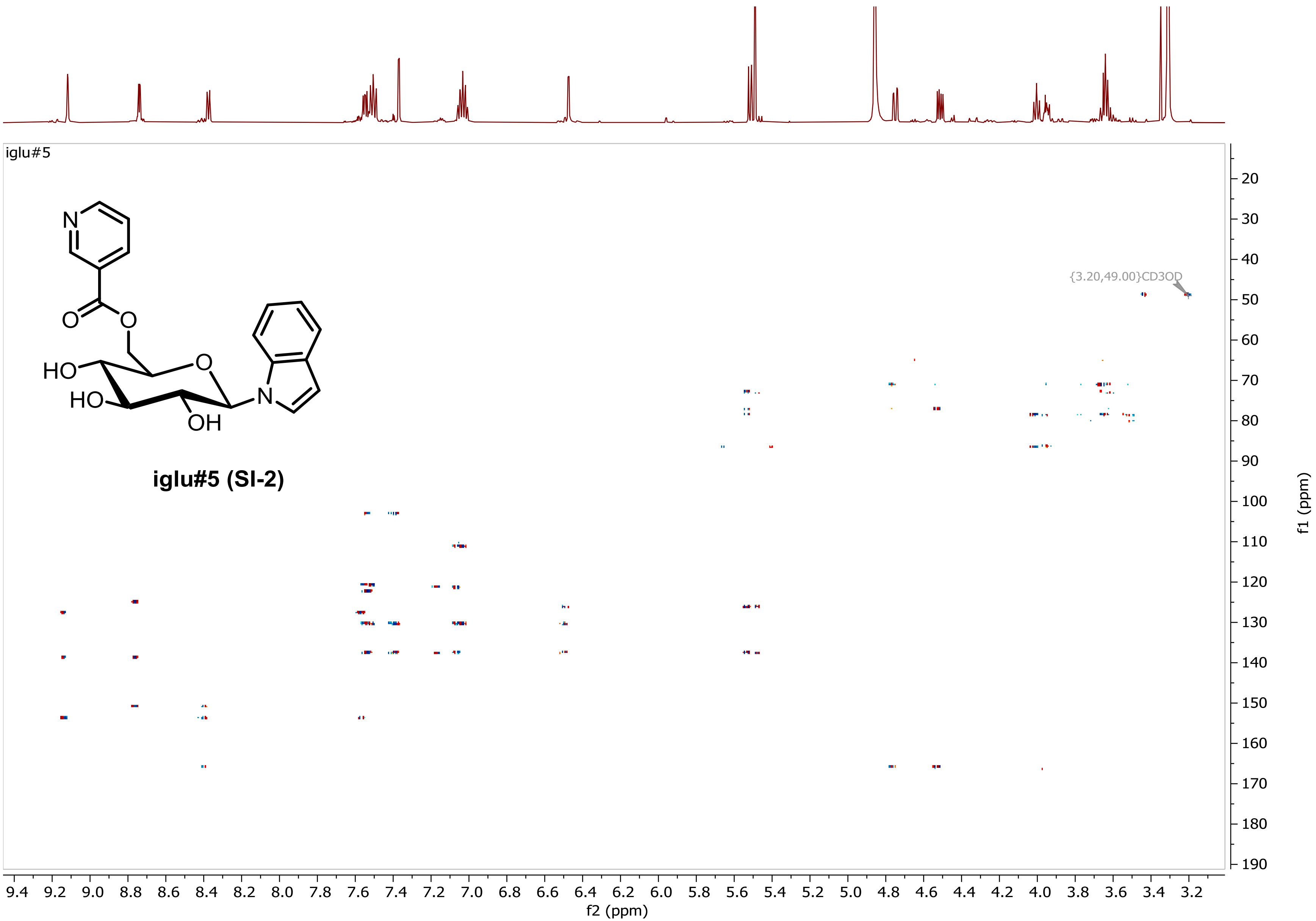

HMBC spectrum (600 MHz) of iglu#5 (**SI-2**) in methanol-d_4.

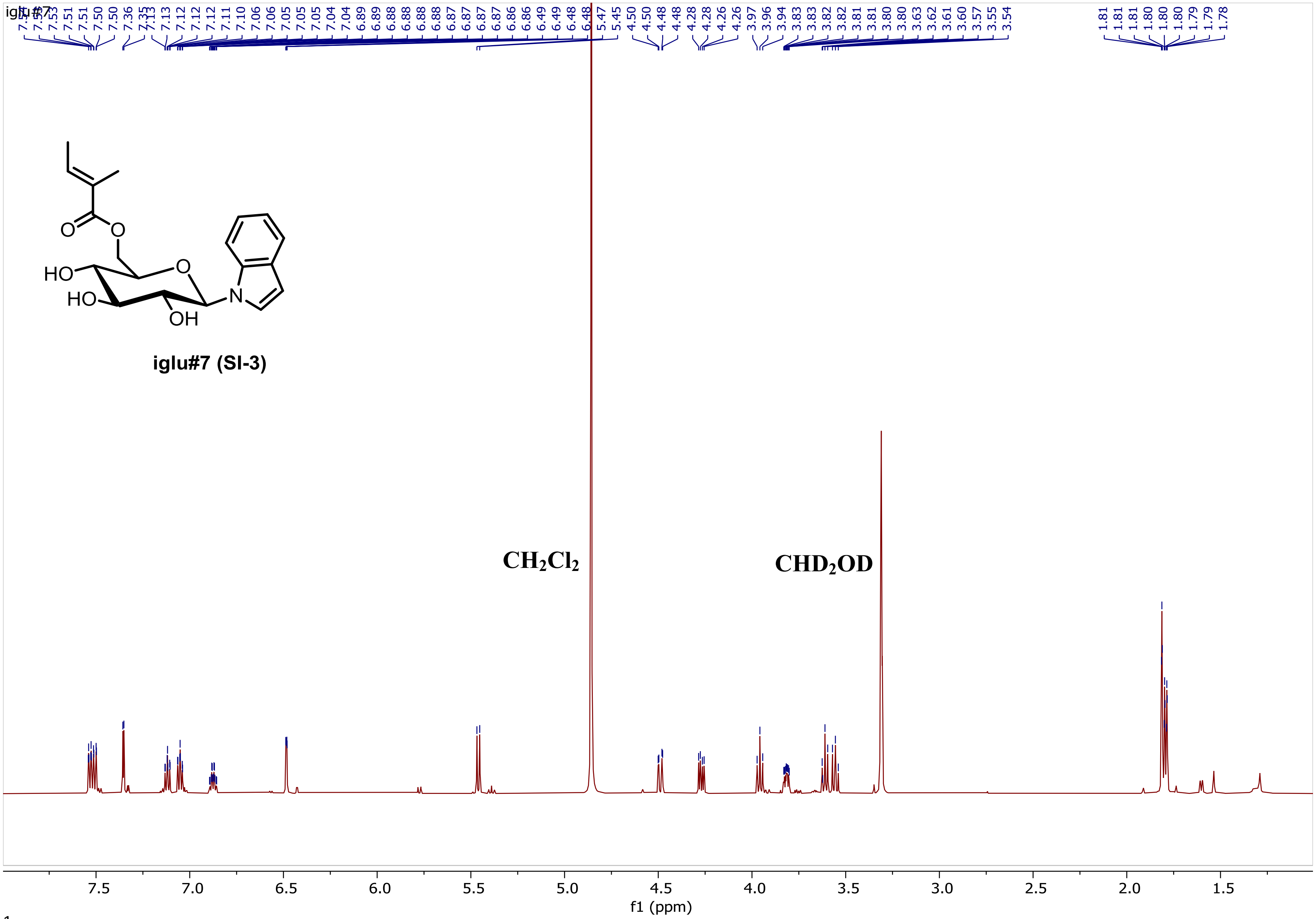

^{1}H NMR spectrum (600 MHz) of iglu#7 (**SI-3**) in methanol-d_4.

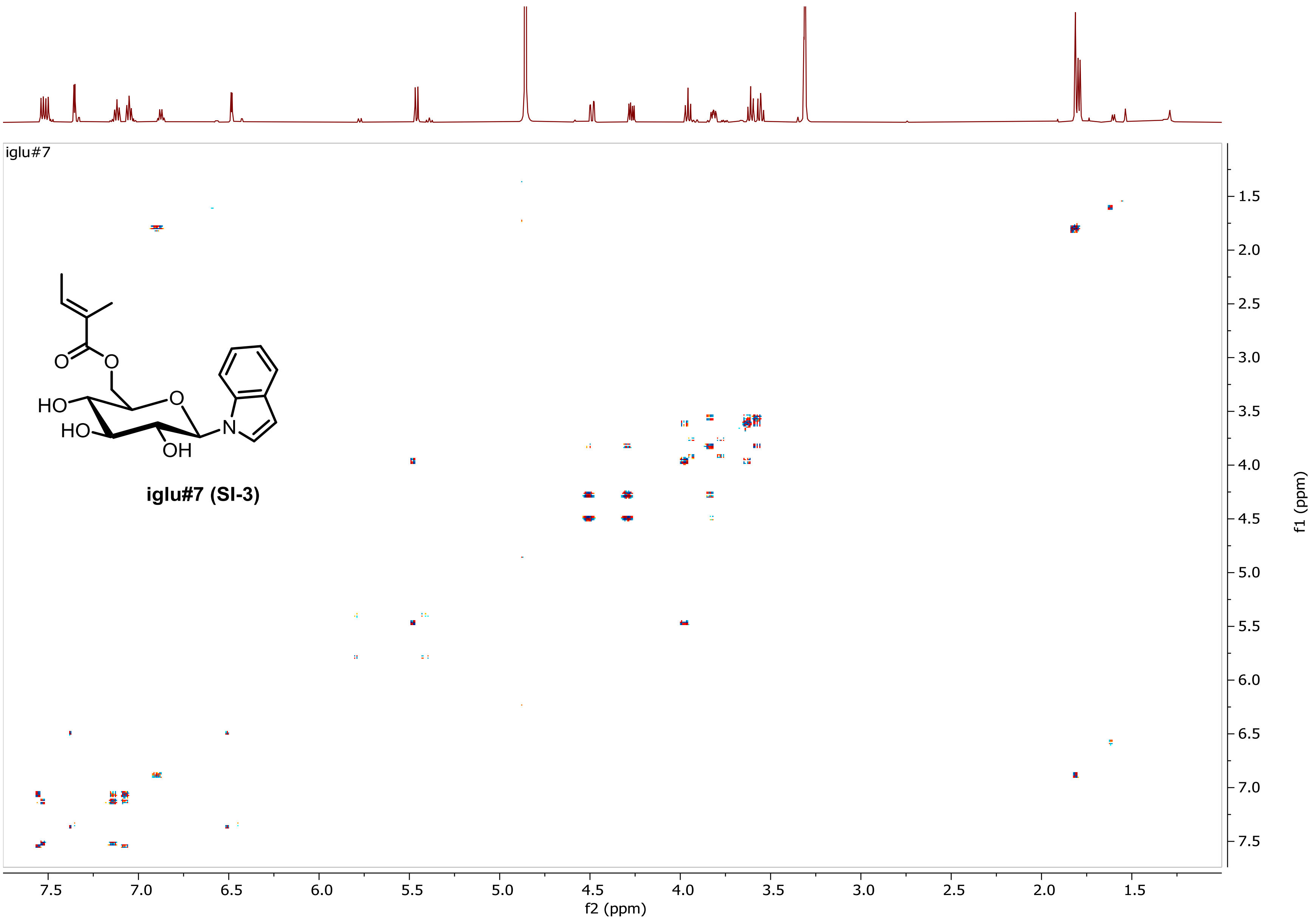

dqfCOSY spectrum (600 MHz) of iglu#7 (**SI-3**) in methanol-d_4.

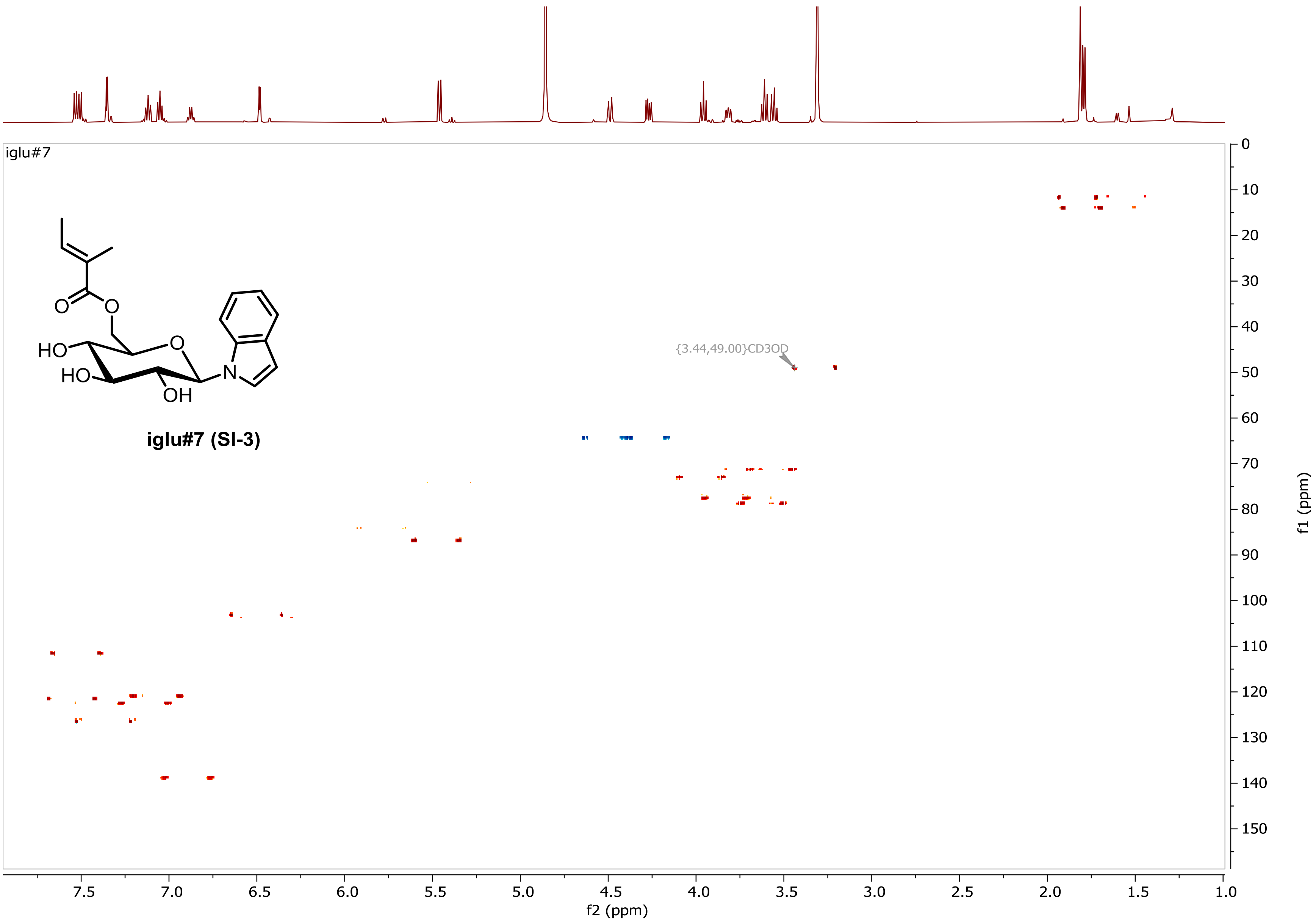

HSQC spectrum (600 MHz) of iglu#7 (**SI-3**) in methanol-d_4.

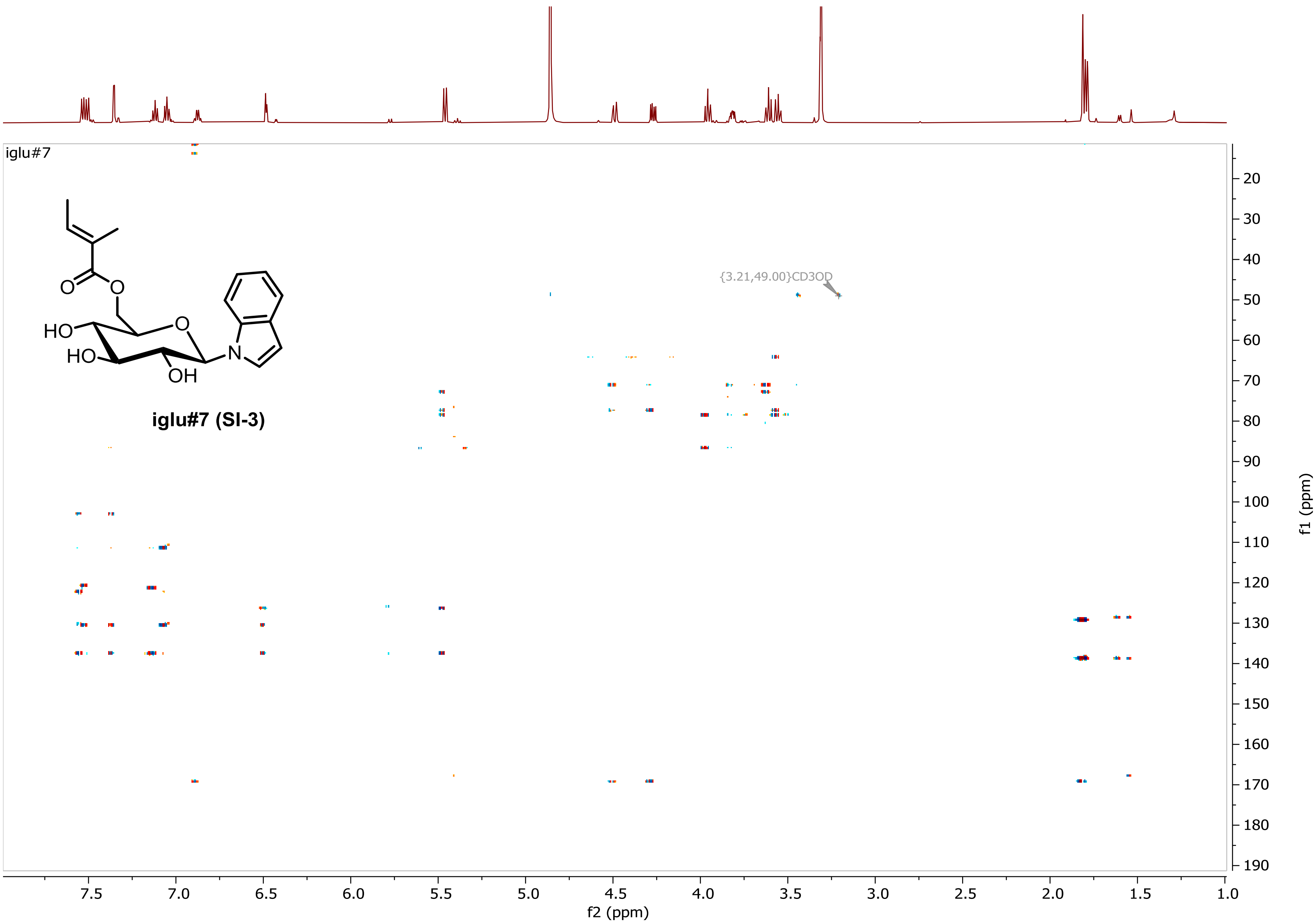

HMBC spectrum (600 MHz) of iglu#7 (**SI-3**) in methanol-d_4.

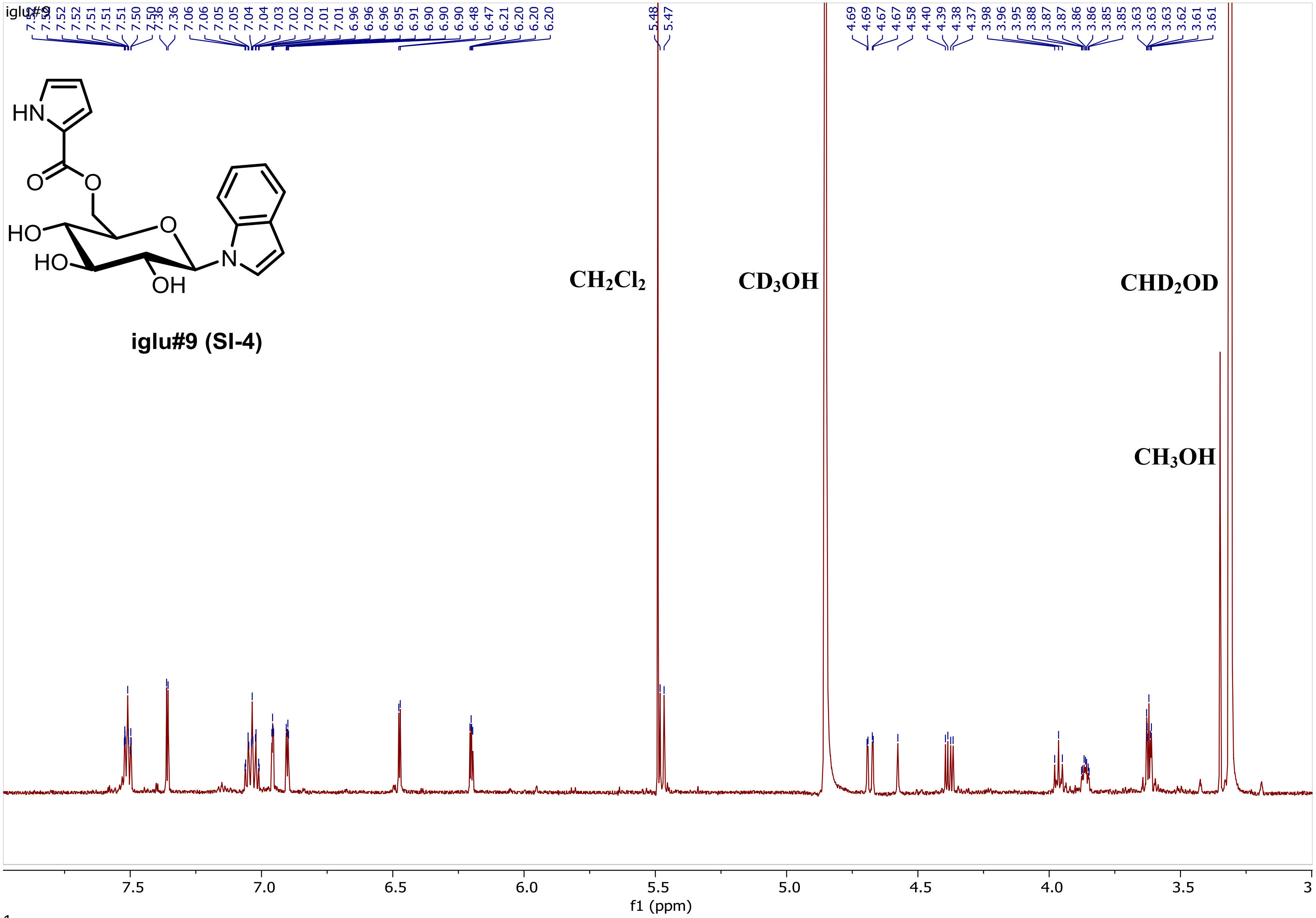

^{1}H NMR spectrum (600 MHz) of iglu#9 (**SI-4**) in methanol-d_4.

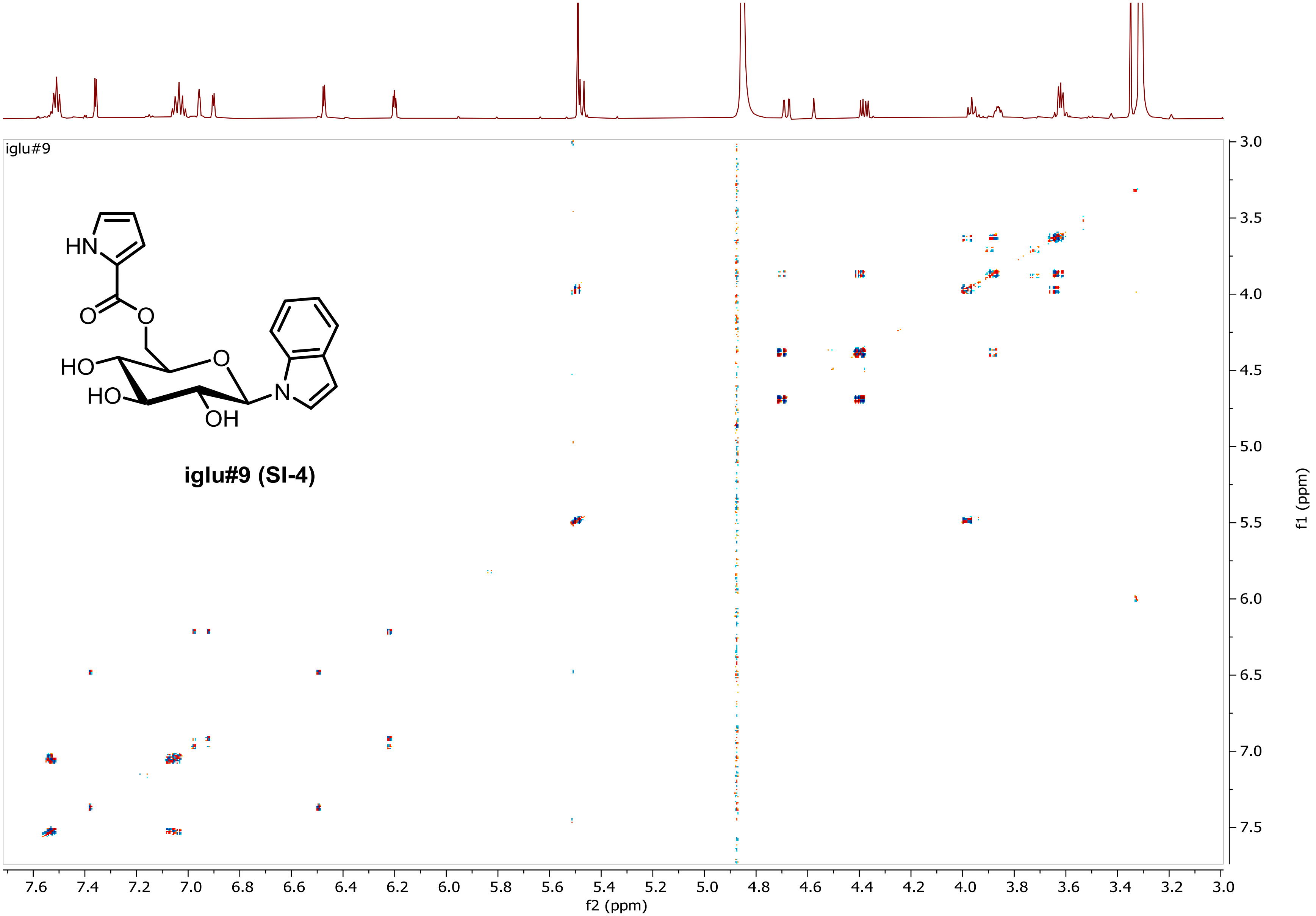

dqfCOSY spectrum (600 MHz) of iglu#9 (**SI-4**) in methanol-d_4.

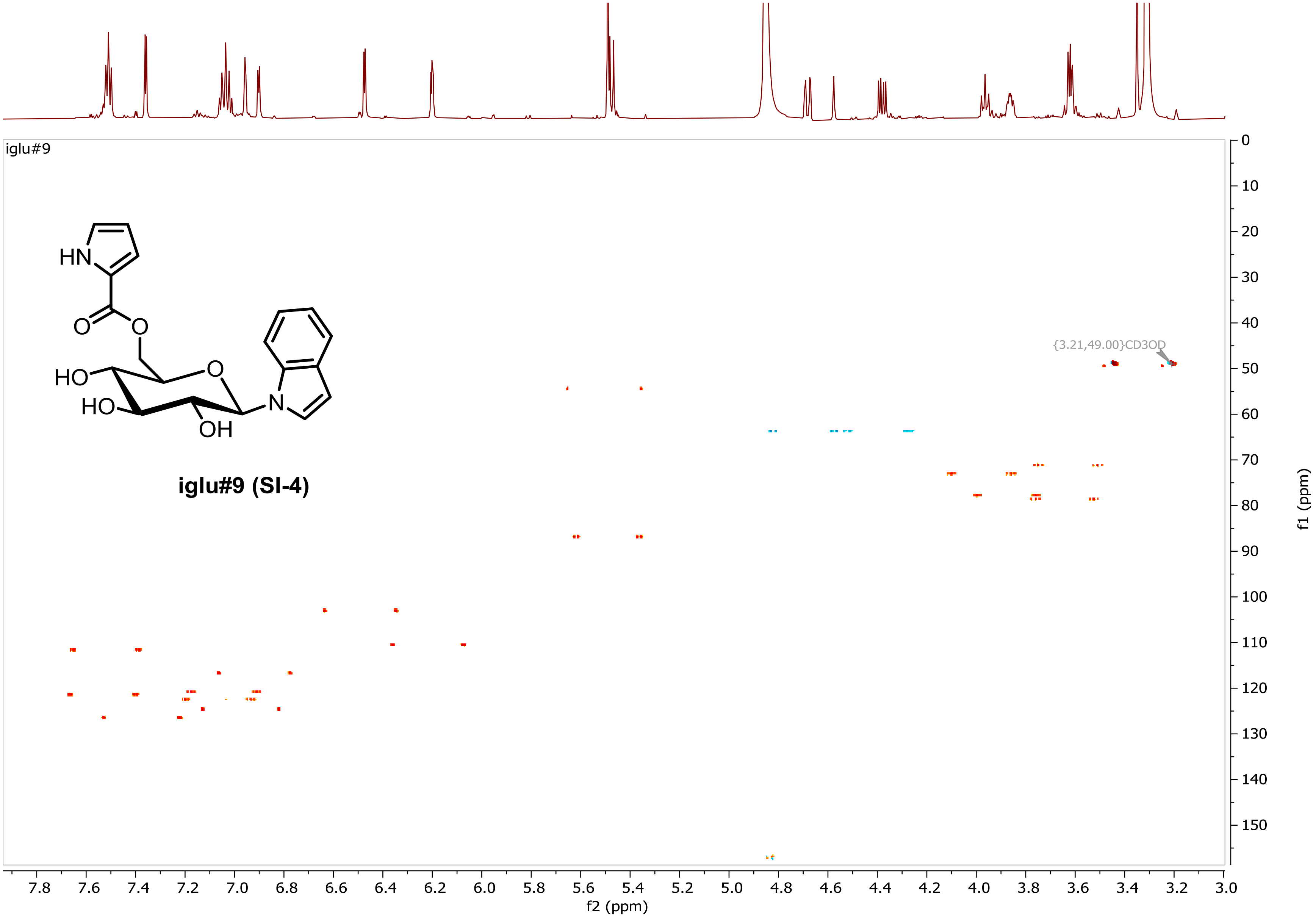

HSQC spectrum (600 MHz) of iglu#9 (**SI-4**) in methanol-d_4.

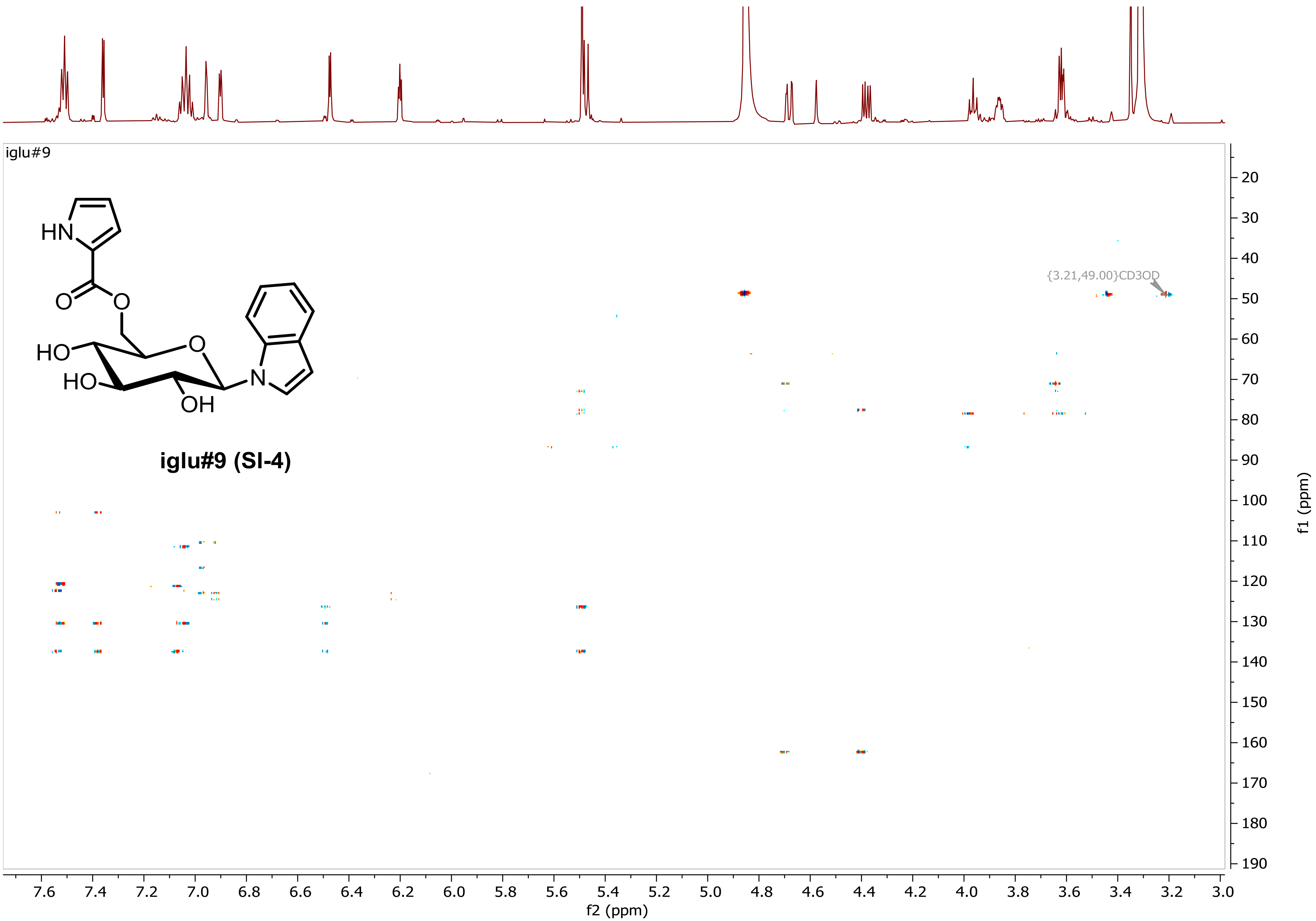

HMBC spectrum (600 MHz) of iglu#9 (**SI-4**) in methanol-d_4.
